# Numerical simulation and parallel implementation of freight train air brake system

**DOI:** 10.1371/journal.pone.0326844

**Published:** 2025-06-25

**Authors:** Zongze Yu, Yuguang Wei, Chuxuan Hu

**Affiliations:** School of Traffic and Transportation, Beijing Jiaotong University, Beijing, China; Beijing University of Technology, CHINA

## Abstract

Numerical simulations of railway air brake systems are becoming increasingly computationally demanding due to the growing complexity and length of trains. This study introduces a parallel computing-enhanced model for simulating pressure dynamics in heavy-haul trains. The proposed approach assigns dedicated threads to each vehicle, with additional threads managing the interface fluxes of brake pipe connections, and is implemented in C# using the ThreadPool and Parallel libraries. A train configuration consisting of one locomotive and 116 vehicles was simulated under various brake pipe pressure reduction conditions. The validity of the air braking system simulation model was confirmed through comparisons with experimental data. Furthermore, the impact of parallel computing on simulation efficiency was investigated. The results indicate that the efficiency of serial computing is primarily influenced by the CPU Boost Clock frequency. Parallel computing consistently outperforms serial computing, with speedup ratios increasing as the number of CPU threads grows. Although the simulation partitions tasks by vehicle units, the computations remain sufficiently fine-grained that the ThreadPool implementation outperforms the Parallel library, as it eliminates the dynamic scheduling overhead inherent to Parallel’s workload distribution mechanism. Additionally, parallel computing efficiency improves as the number of vehicles increases; however, beyond a certain threshold, efficiency stabilizes as workload distribution becomes fully optimized.

## Introduction

**Fig 1:**
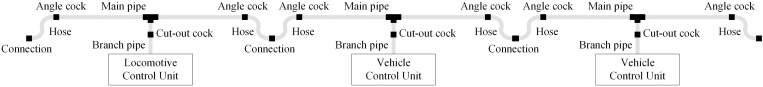
A schematic of a typical air brake configuration.

The air brake system plays a critical role in train braking performance, ensuring safe and efficient deceleration. Typically, as illustrated in Fig 1, an air brake system consists of one or more locomotive and vehicle control units, interconnected by a brake pipe. The locomotive control units regulate the brake pipe pressure, while the vehicle control units manage the charging and discharging of brake cylinders according to pressure variations within the brake pipe.

Despite considerable differences in design and configuration across countries and regions, most vehicle control units are fundamentally based on the well-known Westinghouse triple valve. This valve provides essential functions such as service braking, release, and lapping. By integrating additional valves and reservoirs, the system can also achieve advanced functions like quick service, quick release, and graduated release [1].

To obtain an accurate understanding of train braking performance, it is essential to develop and utilize a mathematical model of the railway air braking system. Existing models can be broadly categorized into empirical models and aerodynamic models. Empirical models employ look-up Tables [2,3] or mathematical equations [4–7] to fit the measured characteristics of air brake systems. In certain models [8,9], the braking force magnitude is assumed to be constant when the dynamic variations in system pressure are neglected. While empirical models are computationally efficient, their accuracy is often compromised due to the oversimplification of dynamic characteristics.

In contrast, aerodynamic models traditionally regard the airflow within the brake pipe as one-dimensional and unsteady. Most of these models [10–14] are derived from the continuity, momentum, and energy equations of the Navier-Stokes (NS) equations. However, some models [15–18] consider only the first two of these equations, while others [19] incorporate constitutive equations to capture additional physical phenomena. Various numerical methods have been employed to solve the NS equations, including the Finite Difference Method (FDM) [17,20], the Finite Element Method (FEM) [12,19], and the Method of Characteristics (MOC) [13,16].

Another widely used numerical approach in fluid dynamics is the Finite Volume Method (FVM), which demonstrates excellent conservation properties, computational efficiency, and numerical stability. Due to its numerous advantages, FVM has been widely applied in fluid dynamics applications [21,22]. However, its application in railway air braking system simulations remains limited, which may be attributed to the dominance of other numerical methods in traditional railway brake system modelling. Considering its ability to accurately calculate airflow between the brake pipe and the locomotive and vehicle control units, FVM holds significant potential for advancing simulation accuracy and efficiency. Therefore, addressing this research gap by developing and validating FVM-based models for railway air brake systems could significantly contribute to the field.

Vehicle control unit models are typically employed to calculate the airflow between reservoirs, based on the motion of the triple valve. The valve motion models include empirical [17,23], quasi-static [12,13,16], and dynamic models [24], all of which generally yield satisfactory results. However, when valves possess very small orifices, dynamic models tend to provide more accurate predictions due to their capacity to capture rapid transients and fine-scale flow dynamics. Airflow between reservoirs is commonly modelled using orifice models [15–19], which estimate the mass flow rate as a function of upstream and downstream pressures and the orifice area.

Among the available simulation models, aerodynamic models offer the highest accuracy, but this precision comes at the cost of significantly increased computational complexity. Specifically, aerodynamic models can require more than 100 times the computational time compared to empirical models [16]. To alleviate this computational burden, recent studies [18,25] have introduced multiprocessing programming techniques to improve simulation efficiency. Although excessive communication overhead between processing threads can, in some cases, prolong the overall computation time [1], parallel programming has been shown to significantly reduce simulation time in railway air brake system modelling [18,25]. However, despite the evident benefits of parallel programming, existing literature lacks comprehensive analysis regarding the key factors influencing parallel efficiency.

The escalating complexity of railway air brake systems, exemplified by increasingly long train configurations—such as China’s 30,000-tonne trains with 324 vehicles—poses significant challenges for efficient numerical simulations. Capturing airflow and valve dynamics, which govern the charge and discharge of brake cylinders, becomes computationally demanding as train lengths increase. This paper explores the potential of parallel algorithms to enhance simulation efficiency, overcoming the limitations of conventional serial methods. We introduce an aerodynamic model for train air brake system, validated with experimental data from real train tests. Simulations are performed serially and in parallel on three CPUs—Intel Core i5-12600KF, Intel Xeon W-2223, and AMD Ryzen 5 5600G—examining factors such as thread count, Boost Clock frequency, parallel task scheduling overhead, and train length. Our goal is to demonstrate that parallel processing can improve the computational feasibility of analysing these systems, supporting advancements in railway brake design and operational safety.

## Brake pipe model

### Main equations

**Fig 2:**
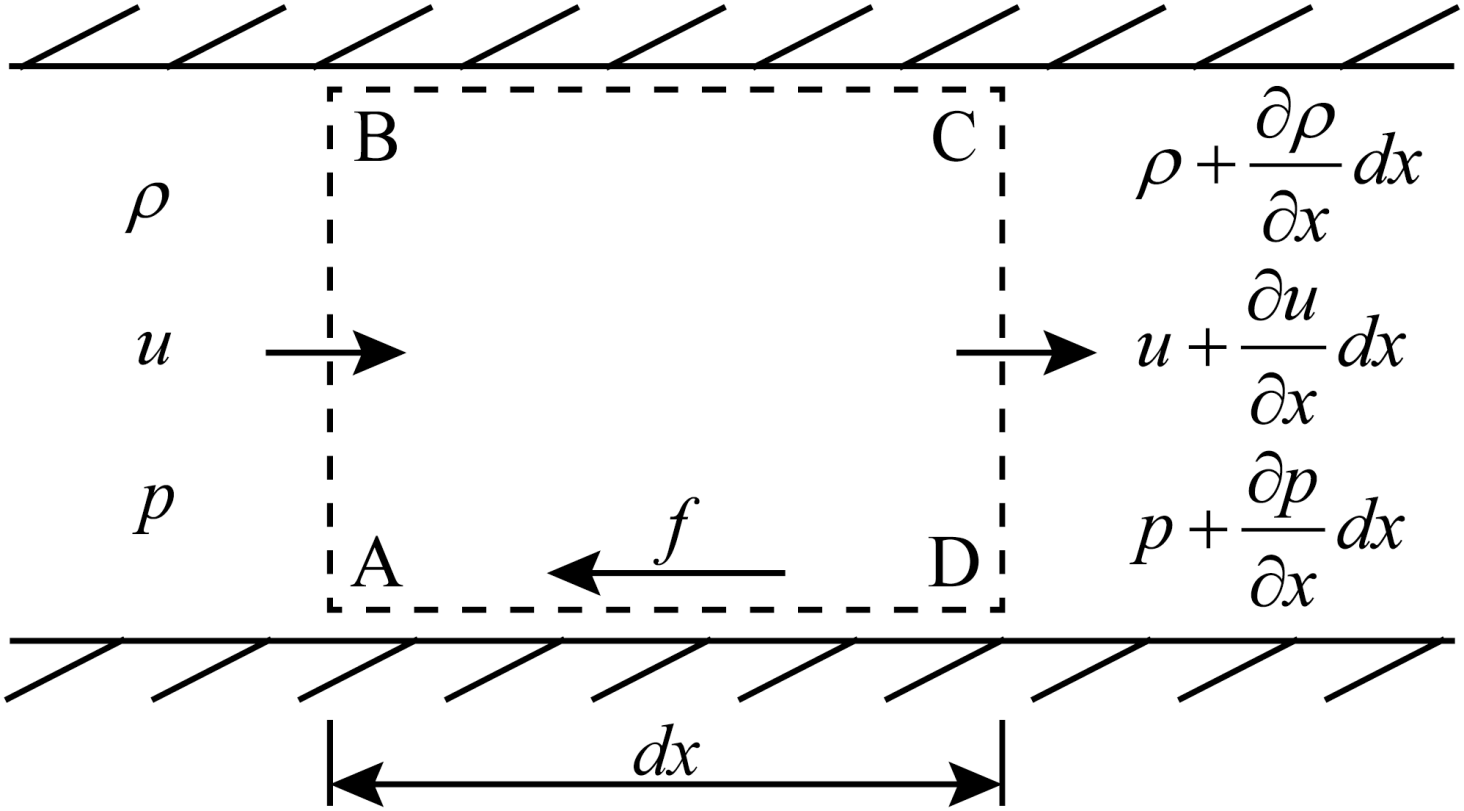
Constant area unsteady flow for brake pipe.


Resolving pressure variations in the brake pipe dominates simulation time due to the computational demands of modelling unsteady airflow across its length. To improve efficiency, we assume a constant cross-sectional area along the pipe, treating variations at connections via local resistances. We further assume that air temperature inside the pipe matches ambient conditions, simplifying thermal effects. These assumptions enable a one-dimensional (1D), unsteady airflow model with uniform cross-section, as depicted in Fig 2. This approach reduces computational overhead while retaining essential dynamics, such as those driving the charge and discharge of brake cylinders. The governing equations for this model, rooted in conservation principles, are detailed as follows:

$$\frac{\partial U}{\partial t}+\frac{\partial F}{\partial x}=S$$ (1)

Equation of continuity


U=ρ,F=ρu,S=0 (2)

Equation of momentum


$$U=\rho u,F=\rho u^{2}+p,S=f$$ (3)

Equation of state


p=ρRT (4)

Equation of constant temperature


$$T=T_{a}$$ (5)

where U is the conserved variables, F is the flux, S is the source term, t is the time variable, x is the position coordinate, ρ is the mass density, u is the velocity, p is the pressure, f is the friction, R is the gas constant, T is the temperature and $T_{a}$ is the ambient temperature.

The friction f can be expressed as.


f=πDτ (6)

with


$$\tau =\frac{1}{8}\mu \rho u^{2}$$ (7)

where D is the diameter of brake pipe, τ the wall shear and μ the friction factor determined by the Reynolds number Re and the equivalent roughness $\frac{\varepsilon }{D}$ [26], which can be defined as


$$\begin{array}{c} \mu =\{\begin{array}{cc} {}*20l\frac{64}{Re}, & ifRe<2300\\ 0.00025\;Re^{\frac{1}{3}}, & if2300\leq Re<4000\\ {\left(2\log_{10}\left(\frac{\varepsilon }{3.7D}+\frac{5.74}{Re^{0.9}}\right)\right)}^{-2}, & ifRe\geq 4000 \end{array} \end{array}$$ (8)

### The FVM

**Fig 3:**
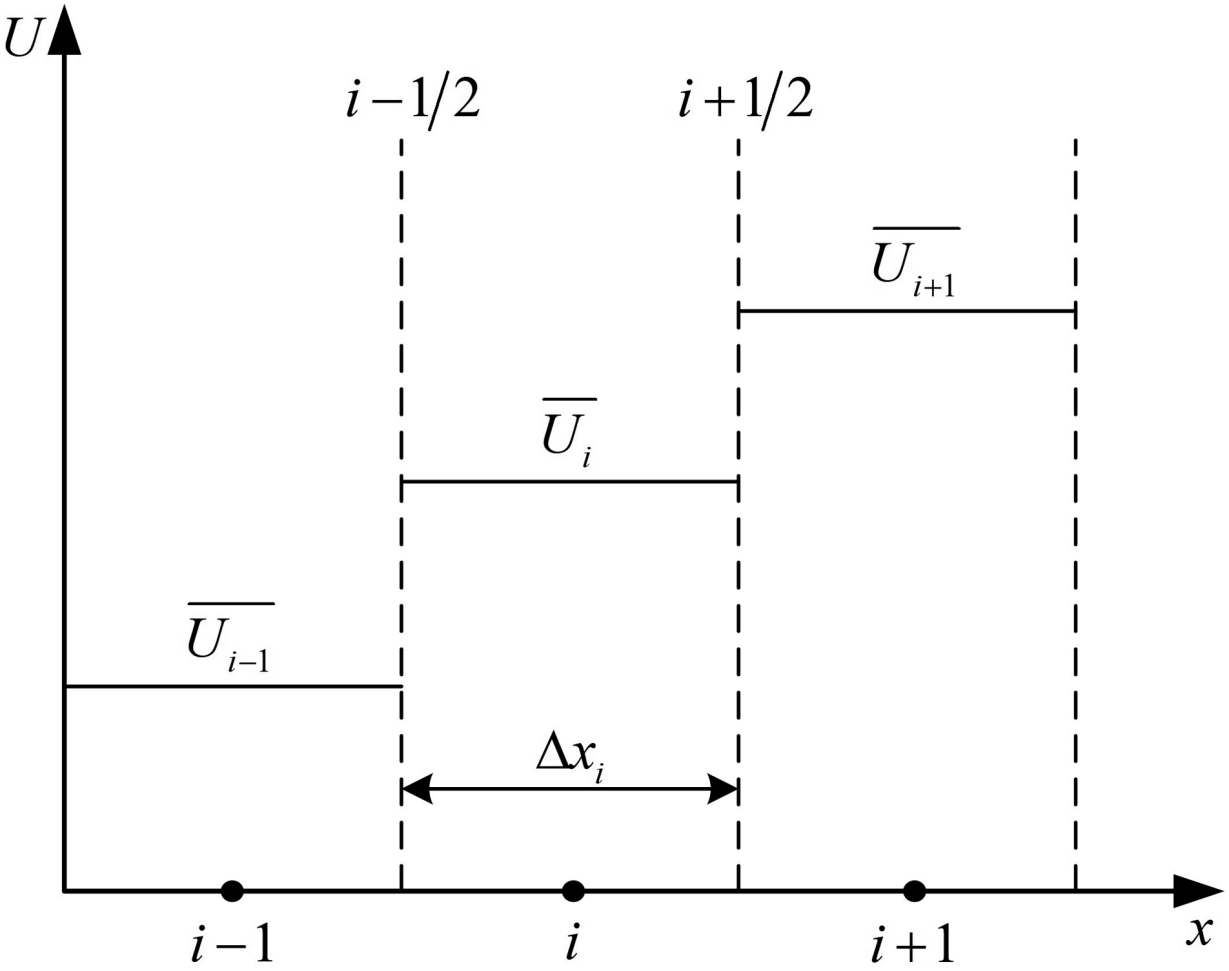
The discrete schematic of FVM.


To solve the governing equations numerically, we discretize the brake pipe into non-overlapping grids using FVM, as illustrated in Fig 3. The FVM is particularly well-suited for modeling the propagation of pressure waves and mass flow in the brake pipe, as it ensures conservation of mass and momentum across control volumes, which is essential for capturing the dynamic behavior of compressible air in long pipelines. This approach ensures accurate integration of the conservation laws over each element, transforming Equation (1) into its discrete form by integrating over element *i*:

dUi―/dUi―dt\nulldelimiterspacedt=(Fi−1/12\nulldelimiterspace2−Fi+1/12\nulldelimiterspace2)/(Fi−1/12\nulldelimiterspace2−Fi+1/12\nulldelimiterspace2)Δxi\nulldelimiterspaceΔxi+Si― (9)

where $\overset{―}{U_{i}}$ and $\overset{―}{S_{i}}$ are the mean conserved variables and source terms within element i, respectively; Fi−1/12\nulldelimiterspace2 and Fi+1/12\nulldelimiterspace2 are the numerical fluxes at the left and right interfaces of element i; and $\Delta x_{i}$ is the length for element i.

A critical step in FVM is computing the interface fluxes. This begins by determining the left and right conserved variables at each interface using values from adjacent grid cells. To ensure stability and avoid numerical oscillations, we adopt the non-oscillatory, non-free-parameter dissipation difference (NND) scheme [27], which effectively captures discontinuities in the flow. The left and right variables at interface i+1/12\nulldelimiterspace2 are calculated as follows:


Ui+1/12\nulldelimiterspace2L=Ui―+12minmod\nolimits(Ui+1―−Ui―,Ui―−Ui−1―) (10)

Ui+1/12\nulldelimiterspace2R=Ui+1―−12minmod\nolimits(Ui+1―−Ui―,Ui+2―−Ui+1―) (11)

The minmod function is defined as:


$$\begin{array}{c} minmod\mathrm{\backslash nolimits}(a_{1},a_{2})=\{\begin{array}{cc} {}*20ls\min\left\{\left|a_{1}|,|a_{2}\right|\right\}, & ifs=sgn(a_{1})=sgn(a_{2})\\ 0, & otherwise \end{array} \end{array}$$ (12)

where Ui+1/12\nulldelimiterspace2L and Ui+1/12\nulldelimiterspace2R represent the left and right conserved variables at the interface i+1/12\nulldelimiterspace2, respectively.

**Fig 4:**
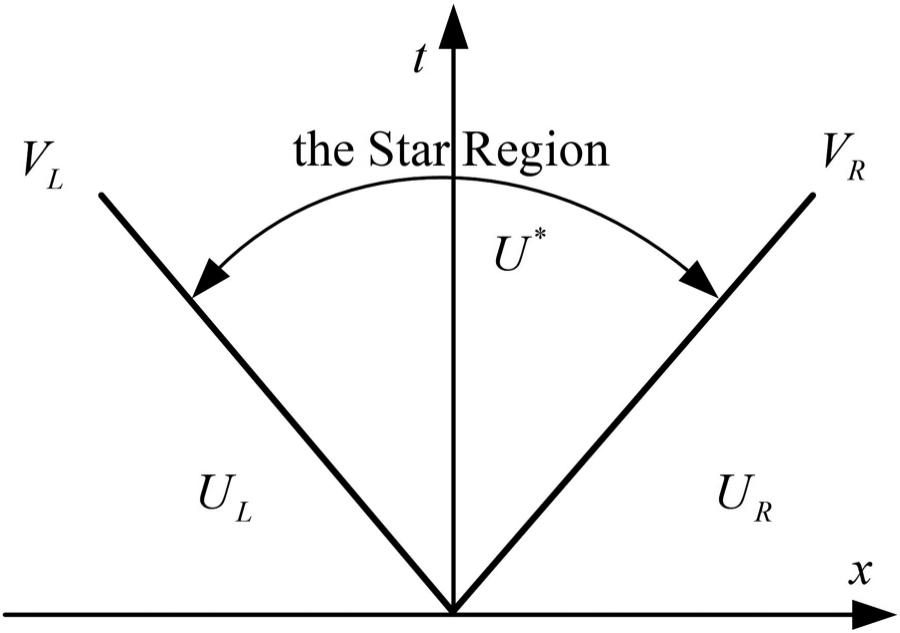
Approximate HLL Riemann solver.

Subsequently, based on Ui+1/12\nulldelimiterspace2L and Ui+1/12\nulldelimiterspace2R, Fi+1/12\nulldelimiterspace2 can be determined, which is essentially a Riemann problem. We adopt the Harten-Lax-van Leer (HLL) Riemann solver, which approximates the wave structure by assuming two waves separating a constant “Star Region,” as shown in Fig 4. The HLL Riemann solver was selected due to its robustness in handling shock-like discontinuities and wavefronts, which naturally occur during rapid brake applications and releases. Unlike more diffusive methods, HLL provides a good balance between accuracy and computational stability, particularly in the presence of wave propagation and non-linear interactions between boundary conditions and pipe segments.

To compute the HLL flux, the following steps are performed [28]:

- *Step I*: Pressure estimate. Compute estimate for the pressure $p_{*}$ in the Star Region as

$$\begin{array}{c} \overset{―}{a}=\frac{1}{2}\left(a_{L}+a_{R}\right),\overset{―}{\rho }=\frac{1}{2}\left(\rho_{L}+\rho_{R}\right),\\ p_{*}=\max\left(0,\frac{1}{2}\left(p_{L}+p_{R}\right)-\frac{1}{2}\left(u_{R}-u_{L}\right)\overset{―}{\rho }\overset{―}{a}\right). \end{array}\}$$ (13)

where $a_{L}$ and $a_{R}$ are the sound speed on the left and right side of the interface, $\rho_{L}$ and $\rho_{R}$ are the sound speeds on the left and right side of the interface, $p_{L}$ and $p_{R}$ are the pressures on the left and right side of the interface, $u_{L}$ and $u_{R}$ are the velocities on the left and right side of the interface respectively.

- *Step II*: Wave speed estimates. Compute the wave speed estimates for $V_{L}$ and $V_{R}$ as

$$V_{L}=u_{L}-a_{L}q_{L},V_{R}=u_{R}+a_{R}q_{R},$$ (14)

with


qK={1ifp*≤pK[1+γ+12γ(p*/p*pK−1\nulldelimiterspacepK−1)]1/12\nulldelimiterspace2ifp*>pK (15)

for K=L and K=R. γ denotes the ratio of specific heats.

- *Step III*: HLL flux. Compute the HLL flux according to

$$\begin{array}{c} F=\left\{ \begin{array}{c} F_{L},\;if0<V_{L}\\ \frac{V_{R}F_{L}-V_{L}F_{R}+V_{L}V_{R}\left(U_{R}-U_{L}\right)}{V_{R}-V_{L}},ifV_{L}\leq 0\leq V_{R}\\ F_{R},\;if0>V_{R} \end{array}\right. \end{array}$$ (16)

where $U_{L}$ and $U_{R}$ are the conserved variables on the left and right side of the interface, $F_{L}$ and $F_{R}$ are the fluxes on the left and right side of the interface respectively.

### Boundary conditions

To accurately model the brake pipe, we implement several boundary conditions following the methodology in [29]:

1. Closed-end boundary: Sets velocity to zero to represent the sealed end of the brake pipe.
2. Partially open boundary: Applies a prescribed pressure to simulate the controllable train tail device (regulating exhaust), the interface between the branch pipe and the upper chamber, or the main reservoir.
3. Adiabatic pressure loss: Introduces a pressure drop to model pipe connections, assuming no heat transfer.
4. Constant pressure at pipe junctions: Maintains a fixed pressure to represent pipe tees.

These conditions ensure realistic simulation of airflow dynamics for the brake pipe. At each boundary, we first estimate the Riemann variables by interpolating values from adjacent grid cells. The interface fluxes are then computed using the HLL Riemann solver, as outlined in [29], ensuring consistency with the interior domain.

## Model of locomotive and vehicle control unit

### Locomotive control unit

**Fig 5:**
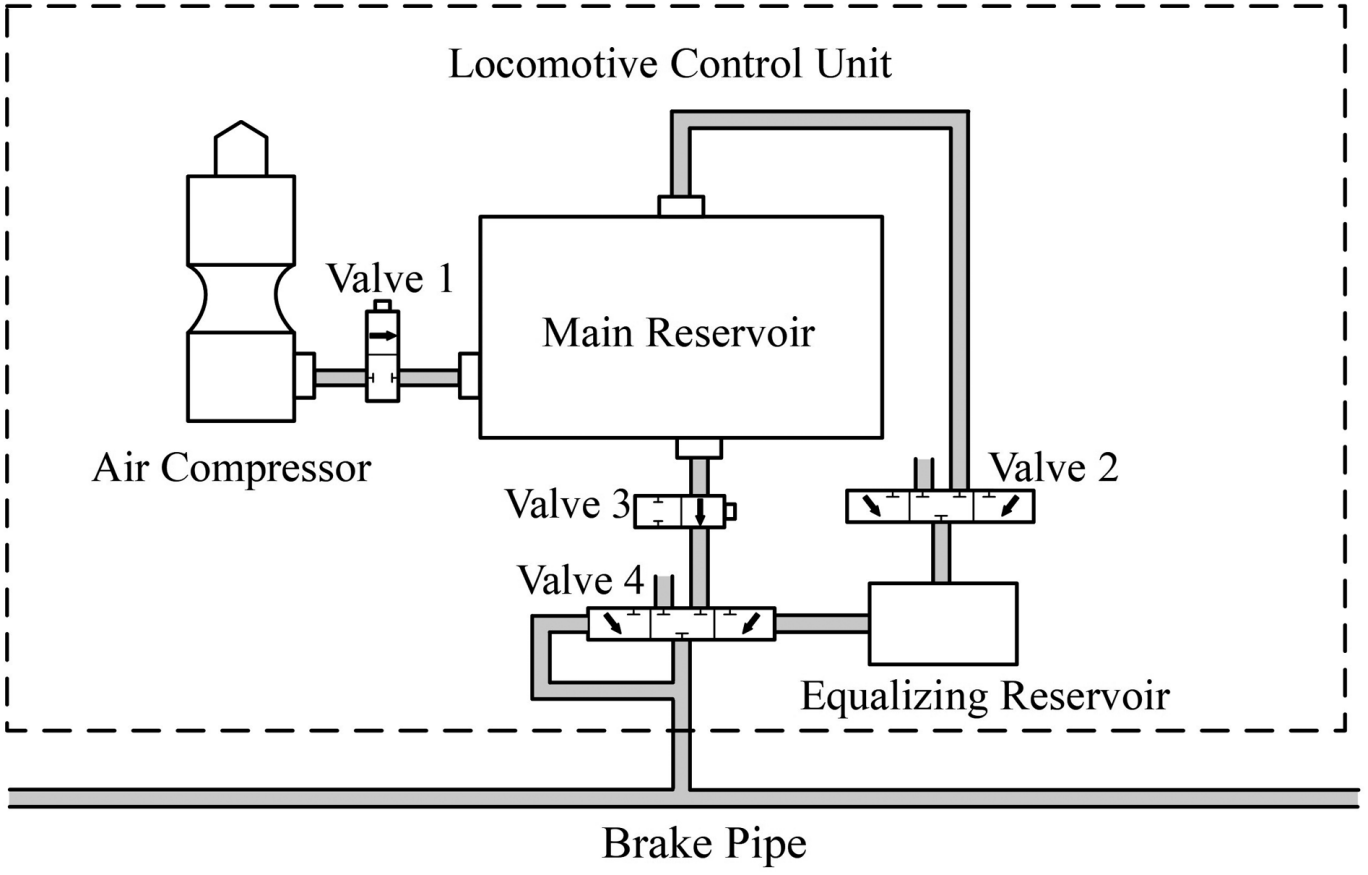
Simplified structure of the locomotive control unit.

The locomotive control unit regulates brake pipe pressure, a critical factor in the charge and discharge of brake cylinders. Fig 5 illustrates its simplified structure, comprising two air compressors, a main reservoir, an equalizing reservoir, and four valves. The system operates as follows:

- Valve 1 (electronic): Activates the compressor to charge the main reservoir, depending on its pressure level.
- Valve 2 (electronic): Adjusts the equalizing reservoir by charging or discharging it, based on the driver’s brake commands.
- Valve 3 (electronic): During braking, it opens only if a pressure hold function is active, preventing significant pressure drops in the brake pipe due to leakage; otherwise, it remains closed.
- Valve 4 (mechanical): Regulates the charge or discharge of the brake pipe, using the equalizing reservoir pressure as the reference.

This model integrates with the brake pipe simulation, enabling accurate prediction of pressure dynamics across the system.

### Vehicle control unit

**Fig 6:**
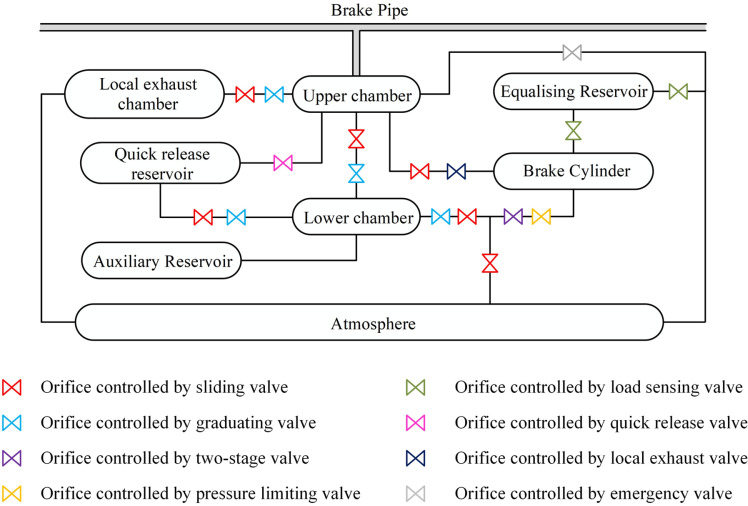
Structure of a China vehicle control unit.

The vehicle control unit in Chinese railway systems adjusts brake cylinder pressure to ensure precise braking. Fig 6 illustrates its structure. Airflow between these reservoirs is regulated by multiple orifices, with their states (open or closed) and areas controlled by valve movements. These mechanisms enable the charge and discharge of the brake cylinder, ensuring effective braking response.

**Fig 7:**
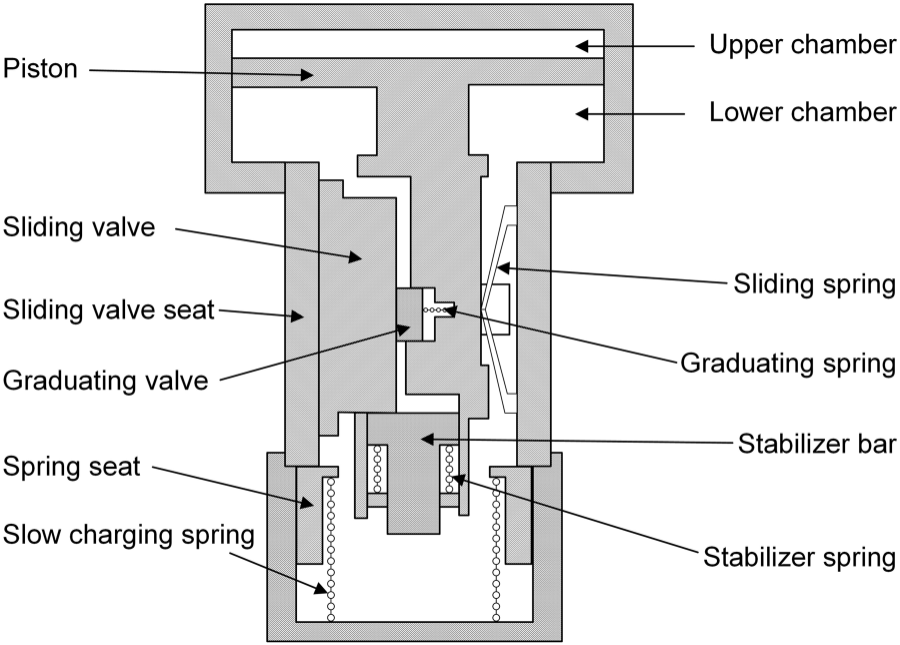
Structure of sliding valve and graduating valve.

The sliding and graduating valves are crucial components in regulating airflow in the vehicle control unit, controlling the charge and discharge of the brake cylinder. Fig 7 illustrates their structure, where a piston, driven by the pressure difference between the upper chamber (connected to the brake pipe) and the lower chamber (linked to the auxiliary reservoir), actuates the valves. The graduating valve is mechanically linked to the piston, while a 6 mm gap between the piston and sliding valve limits their relative motion, ensuring precise control. In the 120 triple valve (a standard in Chinese railways), the sliding valve’s stroke is 14 mm, while the 120−1 variant extends this to 16 mm. The piston’s motion, determined by forces including pressure differences, spring resistance (from sliding and stabilizing springs), and friction, dictates the orifice opening and closing via the positions of the sliding and graduating valves, as supported by the stabilizer bar.

Unlike the sliding and graduating valves, which are modelled dynamically due to their significant motion (as described in the previous section), all other valves in the vehicle control unit—such as quick-release, local exhaust, pressure-limiting, and emergency valves—are modelled using a quasi-static approach. In this method, we assume that these valves respond instantaneously to pressure changes, neglecting transient dynamic effects. The valve position (open or closed) is determined by the equilibrium of forces, primarily the pressure difference across the valve and the spring force, ensuring computational efficiency. This quasi-static model simplifies the simulation of airflow, accurately capturing the charge and discharge of the brake cylinder while reducing computational overhead for these less dynamic components.

### Orifice model for airflow between reservoirs

To simulate the airflow between reservoirs during the charge and discharge processes, we adopt an orifice model based on isentropic flow theory [30]. We assume the reservoir temperatures equal the ambient temperature, simplifying thermal effects. The mass flow rate through the orifice is calculated as:


$$\frac{dm}{dt}=A_{o}C_{m}C_{q}\frac{p_{hi}}{\sqrt{T_{a}}}$$ (17)

with


$$\begin{array}{c} C_{m}=\{\begin{array}{cc} {}*20l\sqrt{\frac{2\gamma }{R(\gamma -1)}\sqrt{{\left(\frac{p_{lo}}{p_{hi}}\right)}^{\frac{2}{\gamma }}-{\left(\frac{p_{lo}}{p_{hi}}\right)}^{\frac{\gamma +1}{\gamma }}}}, & if\frac{p_{lo}}{p_{hi}}\leq b\\ \sqrt{\frac{\gamma }{R}{\left(\frac{2}{\gamma +1}\right)}^{\frac{\gamma +1}{\gamma -1}}}, & if\frac{p_{lo}}{p_{hi}}>b \end{array} \end{array}$$ (18)

and


Cq=0.814−0.1002(plo/plophi\nulldelimiterspacephi)+0.8415(plo/plophi\nulldelimiterspacephi)2−3.9(plo/plophi\nulldelimiterspacephi)3+4.6001(plo/plophi\nulldelimiterspacephi)4−1.6827(plo/plophi\nulldelimiterspacephi)5 (19)

where $A_{o}$ is the equivalent area of orifice, $p_{hi}$ and $p_{low}$ are the high and low pressures of the reservoirs, b is the critical pressure ratio for sonic flow.

The brake cylinder, a key component in applying braking force, features a movable piston, making it a reservoir with a variable volume. As the piston moves—driven by the pressure difference between the brake cylinder and atmosphere, balanced by spring resistance—its displacement alters the cylinder’s volume. To accurately determine the brake cylinder pressure, which directly influences the charge and discharge dynamics, we first calculate the piston’s position using force equilibrium (considering pressure forces and spring resistance). The volume is then updated based on the piston’s displacement. Finally, the pressure is computed using the ideal gas law, integrating with the orifice model for airflow simulation.

## Simulation algorithm

### Time discretization method

To simulate the unsteady airflow and pressure dynamics in the brake pipe and control system, we discretize the time interval into steps. Let $t_{n}$ and $t_{n+1}$ be the current and next time points, where $t_{n+1}=t_{n}+\Delta t$, and Δt is the time step. The aerodynamic models of the brake pipe and control system, previously discretized spatially using FVM, are expressed as a system of ordinary differential equations:


$$\frac{dU}{dt}=Q\left(U\right)$$ (20)

where Q(U) incorporates the flux and source terms from Equation (1) for brake pipe model and mass flow rate from Equation (17) for reservoirs.


U(0)=UnU(1)=U(0)+ΔtQ(U(0))U(2)=3/34\nulldelimiterspace4Un+1/14\nulldelimiterspace4U(1)+1/14\nulldelimiterspace4ΔtQ(U(1))Un+1=1/13\nulldelimiterspace3Un+2/23\nulldelimiterspace3U(2)+2/23\nulldelimiterspace3ΔtQ(U(2)) (21)

The motion of the brake cylinder piston, as well as the slide valve and graduated valve within the control unit, is solved using the Newmark-β method, and the variations in displacement and velocity can be represented as:


$$\begin{array}{c} x_{n+1}=x_{n}+\Delta t\left(\frac{v_{n}+v_{n+1}}{2}\right)\\ v_{n+1}=v_{n}+\Delta t\left(\frac{a_{n}+a_{n+1}}{2}\right) \end{array}$$ (22)

The resulting piston displacement updates the brake cylinder volume, enabling pressure calculations via the ideal gas law, which supports the simulation of charge and discharge dynamics.

### Parallelization strategy

**Fig 8:**
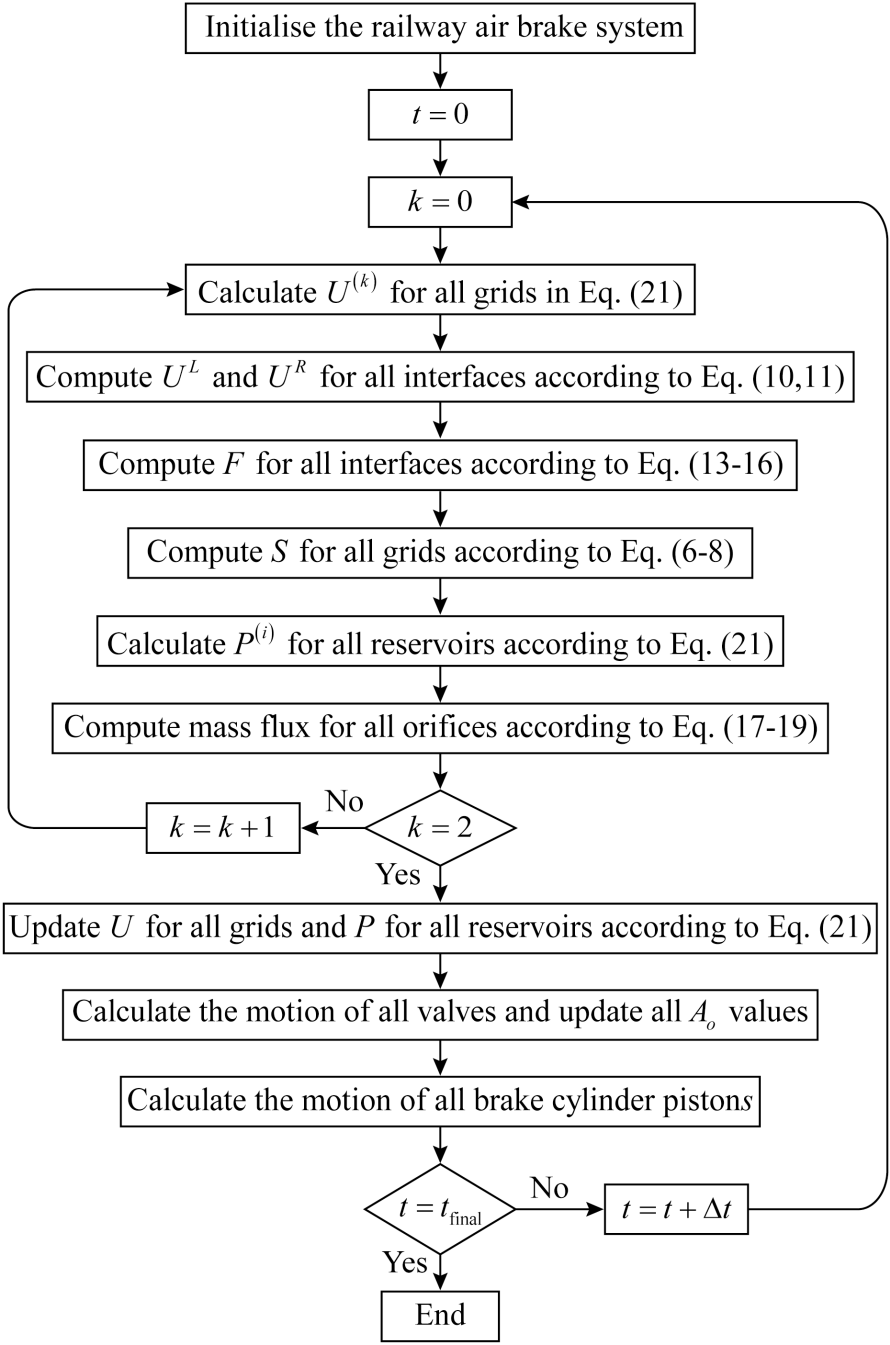
Flowchart for the simulation.

To accelerate simulations of brake pipe and control system dynamics, we parallelize the algorithm in Fig 8. An initial approach assigns individual threads to grid cells for parallel variable updates, leveraging their thread-safe nature and data independence. While this enables fine-grained parallelism, the naive implementation incurs prohibitive overhead from thread management. Thread proliferation not only increases management overhead but also degrades performance through excessive context switching.

Instead, we adopt a coarse-grained approach where each rolling stock unit is processed by a single thread, which manages all associated components: grids, interfaces, orifices, reservoirs, and valves. Interface regions between rolling stock units demand particular attention. For flux calculations at these boundaries (including terminal interfaces), we deploy dedicated threads. All threads synchronize at a barrier before proceeding to the next computation phase.

### Programming language and multi-threading technologies

The simulation algorithm was implemented in C# due to its robust support for parallel programming and compatibility with the.NET framework, which facilitates efficient modeling of brake pipe and control system dynamics. For parallel execution, we considered two approaches to thread management: the Parallel library and the ThreadPool API. The Parallel library, built on top of the.NET ThreadPool, provides automated workload partitioning and dynamic load balancing, making it convenient for general-purpose data-parallel operations. However, it abstracts thread scheduling and reuse, limiting user control over execution details. In contrast, the ThreadPool API allows more direct and fine-grained management of task submission and thread reuse, enabling greater flexibility in coordinating the simulation workflow. These differences in abstraction level and scheduling behavior may influence computational performance under different simulation configurations, which will be analyzed in subsequent sections.

Several prior studies [18,31] have investigated the use of parallel computing to accelerate railway pneumatic brake simulations, with a majority of them employing OpenMP in C or Fortran environments. However, these studies lack detailed disclosure of simulation parameters, which hinders reproducibility and direct comparison. In contrast, the present work is developed entirely in C# on the.NET platform, leveraging its task-based parallelism capabilities. Furthermore, a high-resolution finite-volume model of the brake pipe and control devices is implemented, allowing more accurate representation of pressure wave propagation. To the best of our knowledge, such a.NET-based, detailed pneumatic brake simulation has not been explored in previous literature, making this work a complementary contribution to existing research in the field.

## Simulation results

Building on experimental work [13], we simulated train braking and release processes under two pressure reduction conditions using a train consist with one HXD1 locomotive and 116 C80 freight cars. In the small reduction scenario, we first applied a 50 kPa brake pipe pressure reduction, followed by a second 20 kPa reduction at 74.8 s, with full brake release occurring at 243.7 s. The large reduction scenario involved an initial 50 kPa reduction with subsequent increments totalling 140 kPa, achieving release at 577 s.

**Fig 9:**
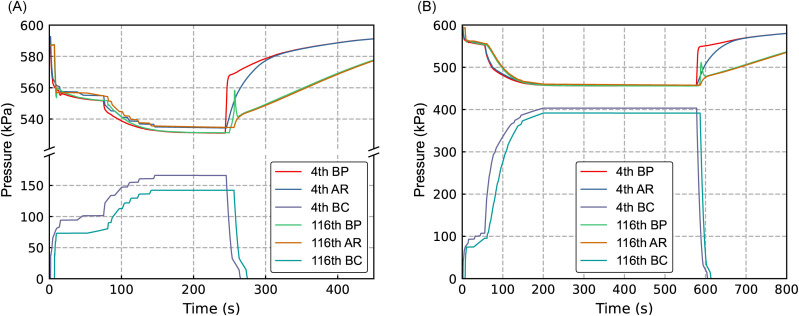
Simulation results for a train consisting of 116 vehicles. (A) Small pressure reduction. (B) Large pressure reduction.

Fig 9 illustrates the simulated pressure variations in the brake pipe (BP), auxiliary reservoir (AR), and brake cylinder (BC) under the given conditions. In the small reduction scenario, the BC pressure of the 4th vehicle increased to 101 kPa following the first reduction and reached 166 kPa after the second, closely matching the experimental value of 165 kPa, with a relative error of 0.6%. Due to the lower AR pressure prior to braking, the stabilized BC pressure of the 116th vehicle was 142 kPa, exhibiting a 3% deviation from the experimental value of 138 kPa. At 450 s, the AR pressure of the 116th vehicle was 578 kPa, differing by only 3 kPa from the experimental measurement of 581 kPa. In the large reduction scenario, the stabilized BC pressures for the 4th and 116th vehicles were 403 kPa and 392 kPa, respectively, corresponding to relative errors of 0.75% and 1% compared to the experimental values of 400 kPa and 388 kPa. Overall, the simulation results demonstrate strong agreement with experimental data, validating the accuracy of the proposed train air braking system model.

**Fig 10:**
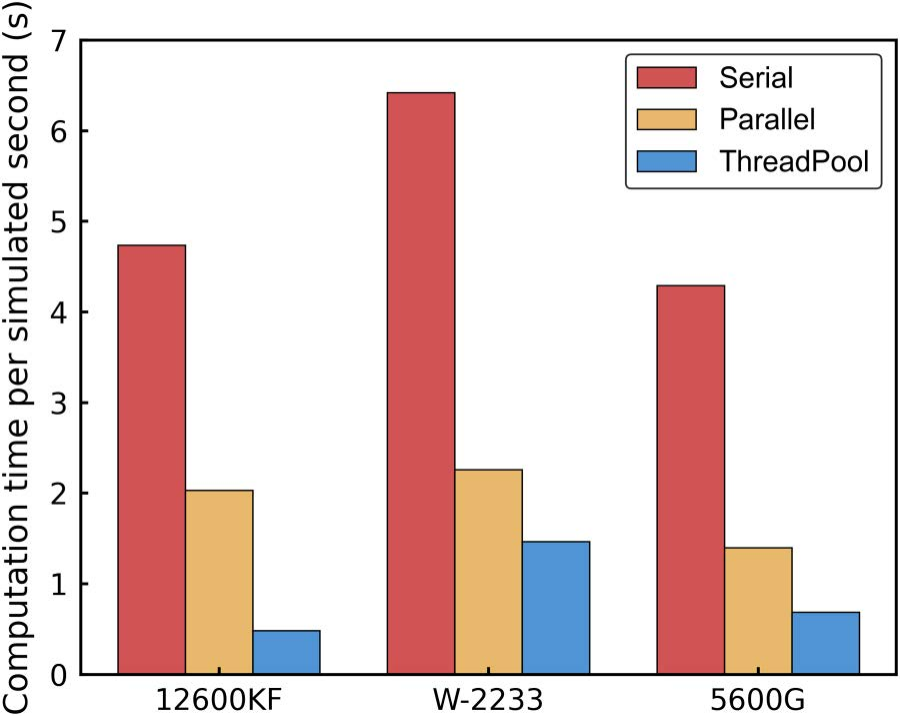
Comparison of CPU simulation efficiency.

Simulations were conducted for the large pressure reduction scenario of the brake pipe, as mentioned above, to evaluate the computational efficiency of serial and parallel implementations. The experiments were performed on three CPUs—Intel Core i5-12600KF, Intel Xeon W-2233, and AMD Ryzen 5 5600G—with their specifications listed in Table 1. Fig 10 presents the average wall-clock time required to simulate one second of real-time operation for the entire train. It is calculated by dividing the total computation time by the total duration of the simulation.

**Table 1:** Specifications of the CPUs.

CPU	Cores	Threads	Base Clock	Boost Clock
Intel Core i5-12600KF	10	16	3.7 GHz/2.8GHz	4.9 GHz/3.6 GHz
Intel Xeon W-2223	4	8	3.6 GHz	4.1 GHz
AMD Ryzen 5-5600G	6	12	3.9 GHz	4.4 GHz

The Boost Clock frequency significantly influenced the performance of the serial implementation. The 5600G, with a Boost Clock of 4.4 GHz, achieved the shortest serial execution time of 4.29 s, whereas the W-2233, with the lowest Boost Clock of 4.1 GHz, recorded the longest at 6.42 s. Despite having the highest Boost Clock of 4.9 GHz on its P-cores, the 12600KF exhibited a serial execution time of 4.74 s, slightly longer than that of the 5600G. This discrepancy is likely due to serial tasks being assigned to its E-cores, which operate at a lower Boost Clock of 3.6 GHz, thereby reducing its effective single-core performance.

For parallel implementations, the number of cores and threads played a critical role in determining the speedup ratio. The 12600KF, with 16 threads, achieved the highest speedup using ThreadPool, reaching a factor of 9.88. In comparison, the 5600G, with 12 threads, attained a speedup of 6.31, while the W-2233, with 8 threads, achieved 4.40, demonstrating that a higher thread count enhances parallel efficiency. The Parallel library exhibited lower performance than the ThreadPool in the simulation, likely due to its internal scheduling and task partitioning strategy. Although the simulation already partitions tasks by vehicle (a coarse-grained approach at the system level), the individual computations per vehicle remain fine-grained—each involving minimal processing time. In such cases, Parallel’s automatic workload distribution can introduce additional overhead from dynamic task splitting, load balancing, and result aggregation. In contrast, the ThreadPool directly queues and executes discrete tasks without intermediate scheduling layers, making it more efficient for fine-grained operations. Thus, despite initial task grouping, Parallel’s inherent overhead for small work units led to ThreadPool’s superior performance. For instance, on the 12600KF, the Parallel library required 2.03 s, whereas ThreadPool completed the computation in only 0.48 s, reflecting a 4.2-fold improvement.

Overall, parallel implementations consistently outperformed serial ones. These results highlight the advantages of parallel algorithms in leveraging multi-core architectures to accelerate large-scale simulations.

**Fig 11:**
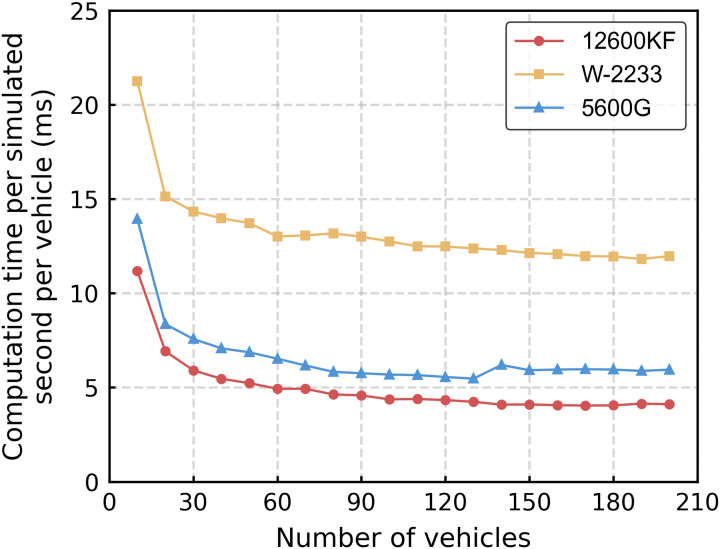
Comparison of simulation efficiency for different numbers of vehicles.

Simulations were conducted on trains with varying numbers of vehicles to evaluate the parallel computing efficiency of the ThreadPool library. The brake pipe pressure was initially set to 600 kPa and reduced by 50 kPa, with the simulation terminating when the brake pipe pressure of the last vehicle dropped below 555 kPa and the total duration exceeded 20 seconds. The simulations were executed on W-2233 and 12600KF CPUs. Fig 11 shows the average wall-clock time required to simulate one second of real-time operation per vehicle, as the number of vehicles increases from 10 to 200. This value is obtained by dividing the total computation time by both the number of vehicles and the total simulated duration.

The results indicate that parallel computing efficiency improves as the number of vehicles increases, primarily due to a more balanced distribution of thread workloads. This improvement is particularly pronounced when the number of vehicles is relatively low. When the number of vehicles increased from 10 to 30, the computational time per vehicle per second decreased by 47%, 33%, and 46% for the 12600KF, W-2233, and 5600G processors respectively. From 30 to 80 vehicles, the reductions were 22%, 8%, and 23%. The improvements became negligible beyond 80 vehicles, with mere reductions of 11%, 9%, and 2% observed when scaling from 80 to 200 vehicles. It is noteworthy that we observed a decrease in computational efficiency when scaling from 130 to 140 vehicles on the 5600G processor. This degradation may be attributed to: (1) the CPU’s limited physical and logical thread capacity constraining parallelization potential, and (2) escalating thread management overhead with increasing vehicle counts.

The findings demonstrate that multithreading technology substantially improves the computational efficiency of railway air brake system simulations. In general, performance scales with the number of available CPU threads, as a greater thread count facilitates more effective utilization of multi-core architectures. Furthermore, parallel computing efficiency increases as the number of vehicles in the train rises; however, this improvement gradually diminishes and stabilizes when the vehicle count becomes sufficiently large.

These trends are particularly relevant in the context of railway freight operations in China. Ordinary freight trains typically consist of approximately 60 vehicles, while heavy-haul trains on dedicated lines such as the Daqin and Shuohuang railways may exceed 200 vehicles per train. The results suggest that parallel acceleration is most beneficial for typical freight train sizes, as it significantly improves computational efficiency in this range. For heavy-haul trains with over 200 vehicles, although further parallelization yields diminishing returns, the simulation still benefits from substantial cumulative time savings due to the large overall problem size. Therefore, the adoption of parallel computing strategies is not only computationally advantageous but also essential for enabling efficient, scalable simulations of complex air brake dynamics in freight and heavy-haul railway systems.

## Conclusions

In this study, we developed a numerical model of a railway air braking system, introduced a parallelization strategy utilizing the ThreadPool and Parallel libraries, and conducted simulations to examine both pressure dynamics and computational efficiency. The accuracy of the model was validated by simulating a train with 116 vehicles under small and large pressure reduction scenarios, with results compared to experimental data. Additionally, parallel computing experiments were performed on three different CPUs (Intel Core i5-12600KF, Intel Xeon W-2233, and AMD Ryzen 5-5600G) and across varying train lengths (10–200 vehicles). Based on these investigations, the following conclusions were drawn:

1. The numerical model effectively captures the pressure dynamics of the BP, AR, and BC during braking and release processes. Under small and large pressure reductions, the simulated BC pressures for the 4th vehicle reached 166 kPa (error: 0.6%) and 403 kPa (error: 0.75%), respectively, while those for the 116th vehicle reached 142 kPa (error: 3%) and 392 kPa (error: 1%). These results closely align with experimental measurements.
2. In the simulation of the train air brake system, the ThreadPool demonstrated superior performance to the Parallel library by a factor of 4.2, as its direct task scheduling proved more efficient for fine-grained computational tasks where Parallel’s dynamic workload distribution incurred disproportionate overhead despite initial task batching by vehicle units.
3. Parallel computing efficiency increases with the number of CPU threads. This is demonstrated by speedup ratios of 9.88 for the 12600KF (16 threads), 6.31 for the 5600G (12 threads), and 4.40 for the W-2233 (8 threads). Parallel implementations consistently outperform serial ones by leveraging multi-core architectures.
4. Parallel computing efficiency improves with the number of vehicles, with a pronounced 38% reduction in computation time on the 12600KF from 10 to 20 vehicles. However, the improvement slows beyond 80 vehicles, with a 11% reduction from 80 to 200 vehicles, indicating stabilization as thread workloads become fully balanced.

In summary, this study demonstrates the effectiveness of parallel implementations in railway air braking system simulations, particularly for heavy-haul trains with a large number of vehicles. The widespread adoption of multi-core processors, heterogeneous computing architectures, and the maturation of cluster/cloud computing technologies, alongside advancements in high-speed network communications, have collectively enhanced parallel computing’s capability to efficiently utilize hardware resources and facilitate large-scale distributed computing. As a result, parallel computing is poised to become the future paradigm for train air braking system simulations. Future investigations will evaluate the parallel algorithms’ performance on server-grade processors featuring enhanced core/thread configurations. Furthermore, the influence of brake pipe modelling assumptions and numerical solution methodologies on both the accuracy and computational efficiency of freight train air brake simulations represents a critical research direction worthy of rigorous examination.

## Supporting information

S1 Table Raw data for Fig 9(A). (XLSX)

S2 Table Raw data for Fig 9(B). (XLSX)

S3 Table Raw data for Fig 10. (XLSX)

S4 Table Raw data for Fig 11. (XLSX)
